# Expression and Subcellular Localization of Mammalian Formin Fhod3 in the Embryonic and Adult Heart

**DOI:** 10.1371/journal.pone.0034765

**Published:** 2012-04-11

**Authors:** Meikun Kan-o, Ryu Takeya, Kenichiro Taniguchi, Yoshihisa Tanoue, Ryuji Tominaga, Hideki Sumimoto

**Affiliations:** 1 Department of Biochemistry, Graduate School of Medical Sciences, Kyushu University, Fukuoka, Japan; 2 Department of Cardiovascular Surgery, Graduate School of Medical Sciences, Kyushu University, Fukuoka, Japan; West Virginia University, United States of Amierca

## Abstract

The formin family proteins play pivotal roles in actin filament assembly via the FH2 domain. The mammalian formin Fhod3 is highly expressed in the heart, and its mRNA in the adult heart contains exons 11, 12, and 25, which are absent from non-muscle Fhod3 isoforms. In cultured neonatal cardiomyocytes, Fhod3 localizes to the middle of the sarcomere and appears to function in its organization, although it is suggested that Fhod3 localizes differently in the adult heart. Here we show, using immunohistochemical analysis with three different antibodies, each recognizing distinct regions of Fhod3, that Fhod3 localizes as two closely spaced bands in middle of the sarcomere in both embryonic and adult hearts. The bands are adjacent to the M-line that crosslinks thick myosin filaments at the center of a sarcomere but distant from the Z-line that forms the boundary of the sarcomere, which localization is the same as that observed in cultured cardiomyocytes. Detailed immunohistochemical and immuno-electron microscopic analyses reveal that Fhod3 localizes not at the pointed ends of thin actin filaments but to a more peripheral zone, where thin filaments overlap with thick myosin filaments. We also demonstrate that the embryonic heart of mice specifically expresses the Fhod3 mRNA isoform harboring the three alternative exons, and that the characteristic localization of Fhod3 in the sarcomere does not require a region encoded by exon 25, in contrast to an essential role of exons 11 and 12. Furthermore, the exon 25-encoded region appears to be dispensable for actin-organizing activities both *in vivo* and *in vitro*, albeit it is inserted in the catalytic FH2 domain.

## Introduction

The sarcomere, the contractile unit of striated muscle, occurs from a direct result of the precise alignment and organized integration of actin-based thin filaments and myosin-based thick filaments [1]. The actin thin filaments, having a uniform length, are tethered at the barbed ends by sarcomeric α-actinin to the Z-line, which forms the boundary of the sarcomere. The pointed ends of the actin filaments are capped with tropomodulin (Tmod), and slide into a lattice of thick myosin filaments that are corsslinked at the M-line in the center of the sarcomere; a zone containing myosin filaments is known as A-band. The highly ordered array of thin actin filaments is not static as previously thought, but dynamically organized during both assembly and maintenance of the sarcomere [2]–[4], although the precise mechanism for organization and turnover of actin filaments in the sarcomere is still unclear [5]. Several actin-associating proteins have recently been proposed as key regulator of actin dynamics during sarcomere organization, for example, the Tmod-related protein leiomodin in cardiomyocytes [6] and the giant actin-binding protein nebulin in the skeletal muscle [7]. Fhod3, a member of formin family proteins that regulate actin filament assembly via the formin homology 2 (FH2) domain [8], [9], is another probable candidate for a key regulator: depletion of Fhod3 in cultured cardiomyocytes disrupts sarcomere organization [10].

**Figure 1:**
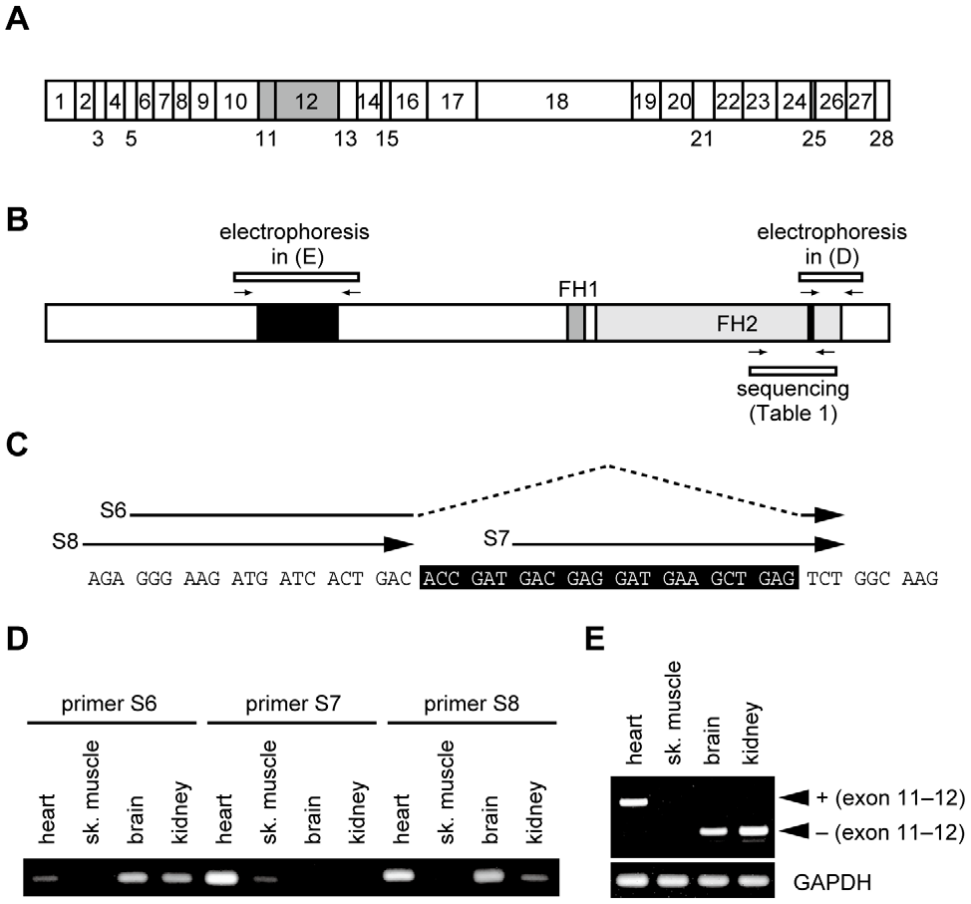
Expression of Fhod3 isoforms in the embryonic tissues. (A) Exon structure of the moue Fhod3 gene. Alternative splicing exons are indicated by gray boxes. (B) Schematic presentation of domain structure of mouse Fhod3. The alternative splicing regions are in black boxes, and primers for RT-PCR analysis are indicated by arrows. (C) Specific primers designed for isoforms derived from the alternative splicing in the C-terminal FH2 domain. The primers ‘S6’ and ‘S7’ are specific for Fhod3 mRNAs with and without exon 25 that encodes the T(D/E)_5_XE region (boxed in black), respectively. The primer ‘S8’ is common for both variants. (D) Tissue-specific expression of the “T(D/E)_5_XE” exon in mouse embryos. The RT-PCR products using specific primers (shown in B and C) were subjected to agarose-gel electrophoresis. sk. muscle, skeletal muscle. (E) Tissue-specific expression of splicing variants lacking exons 11 and 12. The RT-PCR products (shown in B) were subjected to agarose-gel electrophoresis.

The mouse *FHOD3* gene consists of 28 exons (see Figure 1A). As we have previously shown [11], exons 11 and 12 exist in the mRNA expressed in the heart, but are simultaneously spliced out in the kidney and brain. Iskratsch *et al.* have recently reported that the Fhod3 mRNA in the adult heart and skeletal muscle also contains another exon, *i.e.*, exon 25 [12]; this striated muscle-specific exon encodes the acidic-residue-rich sequence of eight amino acids, T(D/E)_5_XE, that is inserted at the position close to the C-terminal end of the FH2 domain. It has remained unclear whether these alternative exons occur during development of the heart.

Using rat neonatal cardiomyocytes in culture, we have previously shown that Fhod3 protein localizes in the middle of the sarcomere as two bands separated by the M-line: they are more adjacent to the M-line than to the Z-line [10]. The characteristic localization of Fhod3 within the sarcomere has recently been confirmed using the same cells [12]. The N-terminal region, which is encoded by exons 11 and 12, appears to play a crucial role in Fhod3 localization, because the Fhod3 isoform lacking this region fails to localize to the sarcomere when ectopically expressed in cultured cardiomyocytes [10]. On the other hand, it is still possible that Fhod3 localization in cultured cardiomyocytes may not reflect *in situ* localization, since the sarcomere in freshly isolated cardiomyocytes once disassembles and then reassembles or newly assembles during culture *in vitro* [4], [13]. Iskratsch *et al.* has recently proposed that, in the adult heart, Fhod3 mainly localizes to the Z-line but not to the middle region of the sarcomere, in contrast to its localization in cultured cardiomyocytes [12].

Here we show that, in sections prepared from embryonic and adult hearts of mice as well as those from an adult human heart, three independent antibodies against Fhod3 all exist as two bands in the middle of the sarcomere in the same manner as in cultured cardiomyocytes. Detailed immunohistochemical studies by co-staining with antibodies against Tmod and immuno-electron microscopic analysis reveal that Fhod3 localizes not at the pointed ends of thin actin filaments but to a more peripheral zone, where thin filaments overlap with thick myosin filaments. We also demonstrate that the Fhod3 mRNA isoform in the heart of mice embryos also contains the alternative exons 11, 12, and 25, and that the characteristic sarcomere localization of Fhod3 is independent of the T(D/E)_5_XE region encoded by exon 25, in contrast to an essential role of exons 11 and 12. Furthermore, the exon 25-encoded region appears to play a dispensable role in actin-assembling activities despite of its localization within the catalytic FH2 domain.

## Results

### Expression of Fhod3 isoforms in embryonic mice

To investigate the Fhod3 isoform expressed in the embryonic heart, we first tested whether exon 25 is present in Fhod3 mRNAs by RT-PCR analysis with specific primers (Figures 1B and 1C) using total RNA obtained from various tissues of mouse embryos at embryonic day 17.5 (E17.5) (Figure 1D); the presence of exon 25 was confirmed by sequence analysis of RT-PCR products subcloned (Table 1). Fhod3 mRNAs containing exon 25 were expressed highly in the heart and slightly in the skeletal muscle, while exon 25 was absent from the Fhod3 mRNAs in the brain and kidney (Figure 1D).

**Table 1:** Expression of alternatively spliced variants of Fhod3.

	Heart		Skeletal muscle		Brain		Kidney	
Variant type	Adult	Embryo	Adult	Embryo	Adult	Embryo	Adult	Embryo
T(D/E)_5_XE (−)	0 (0)	1 (10)	0 (0)	1 (8)	N.D.*	10 (100)	N.D.	15 (100)
T(D/E)_5_XE (+)	10 (100)	9 (90)	10 (100)	11 (92)	N.D.	0 (0)	N.D.	0 (0)

The RT-PCR fragments amplified using specific primers flanking exon 25 (see Figure 1B) are subcloned and subjected to sequencing analysis. The number in parenthesis indicates the percentage of each variant in the indicated tissue.

* N.D. not determined.

We next investigated the alternative splicing of exons 11 and 12 in Fhod3 mRNAs by RT-PCR with two primers that flank exons 11–12 (Figure 1B) using total RNA from mouse embryonic tissues. As shown in Figure 1E, exons 11 and 12 were spliced out from the Fhod3 mRNAs in the brain and kidney, whereas these two exons were retained in the cardiac isoform. A similar tissue-specific splicing of the Fhod3 mRNAs occurs also in the adult mice [11], [12]. Taken together, the embryonic heart as well as the adult one appears to express almost exclusively the Fhod3 mRNA isoform containing all the three alternative exons 11, 12, and 25.

### Localization of Fhod3 in the embryonic heart

**Figure 2:**
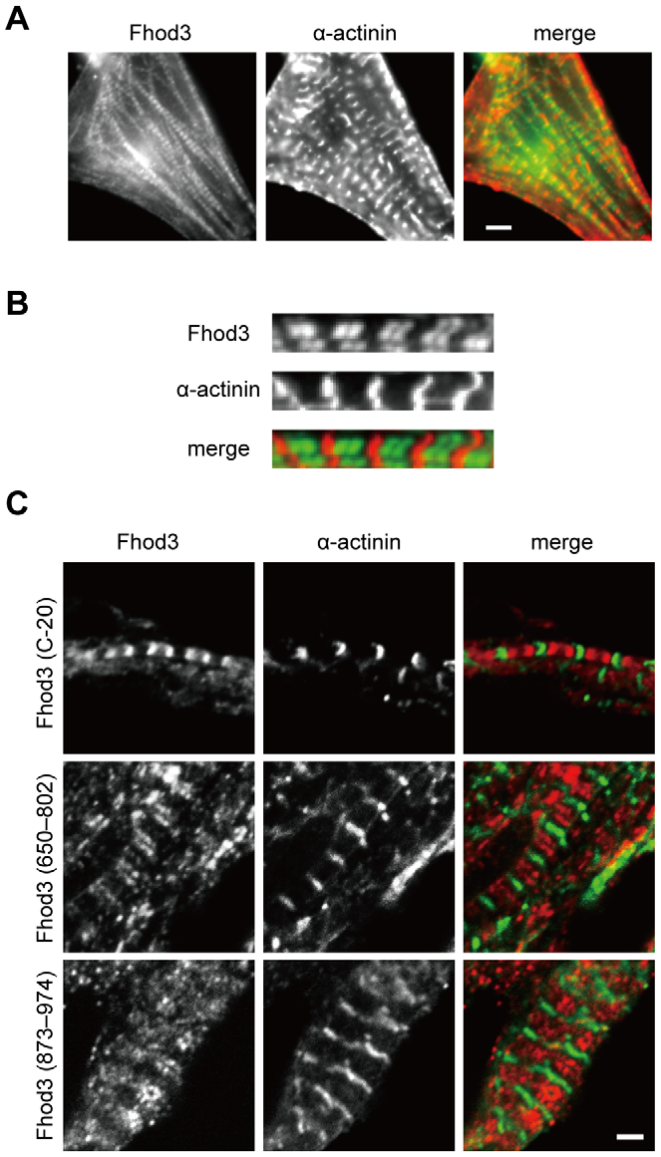
Localization of Fhod3 in the embryonic heart. (A) Embryonic mouse cardiomyocytes were subjected to immunofluorescent double staining for endogenous Fhod3 (green) and α-actinin (red). For Fhod3 staining, the anti-Fhod3-(650–802) polyclonal antibodies were used. Bar, 5 µm. (B) Magnified image of single myofibril from immunostained embryonic mouse cardiomyocytes. (C) Sections of mouse embryonic hearts were subjected to immunofluorescent double staining for endogenous Fhod3 (red) and α-actinin (green). For Fhod3 staining, the anti-Fhod3-(C-20) (top panels), the anti-Fhod3-(650–802) (middle panels), and the anti-Fhod3-(873–974) (bottom panels) polyclonal antibodies were used. Bar, 2 µm.

To investigate Fhod3 localization in the embryonic heart, we first performed immunofluorescence staining with the anti-Fhod3-(650–802) antibodies using cultured cardiomyocytes that were isolated from the mouse embryonic heart. As shown in Figures 2A and B, Fhod3 localized as two closely spaced bands in the sarcomere, which was bordered by the Z-lines marked with the anti α-actinin antibody. The manner of Fhod3 localization in embryonic mouse cardiomyocytes is the same as that observed in rat neonatal cardiomyocytes [10]. The characteristic localization of Fhod3 in the sarcomere was also observed in frozen sections of mouse embryonic hearts, when stained with the anti-Fhod3-(C-20) antibodies (Figure 2C). Essentially the same results were obtained by staining with two different anti-Fhod3 antibodies, *i.e.*, the anti-Fhod3-(650–802) and anti-Fhod3-(873–974) antibodies. Thus Fhod3 appears to predominantly localize *in situ* as double bands in the sarcomere of the mouse embryonic heart.

### Localization of Fhod3 in the adult heart

**Figure 3:**
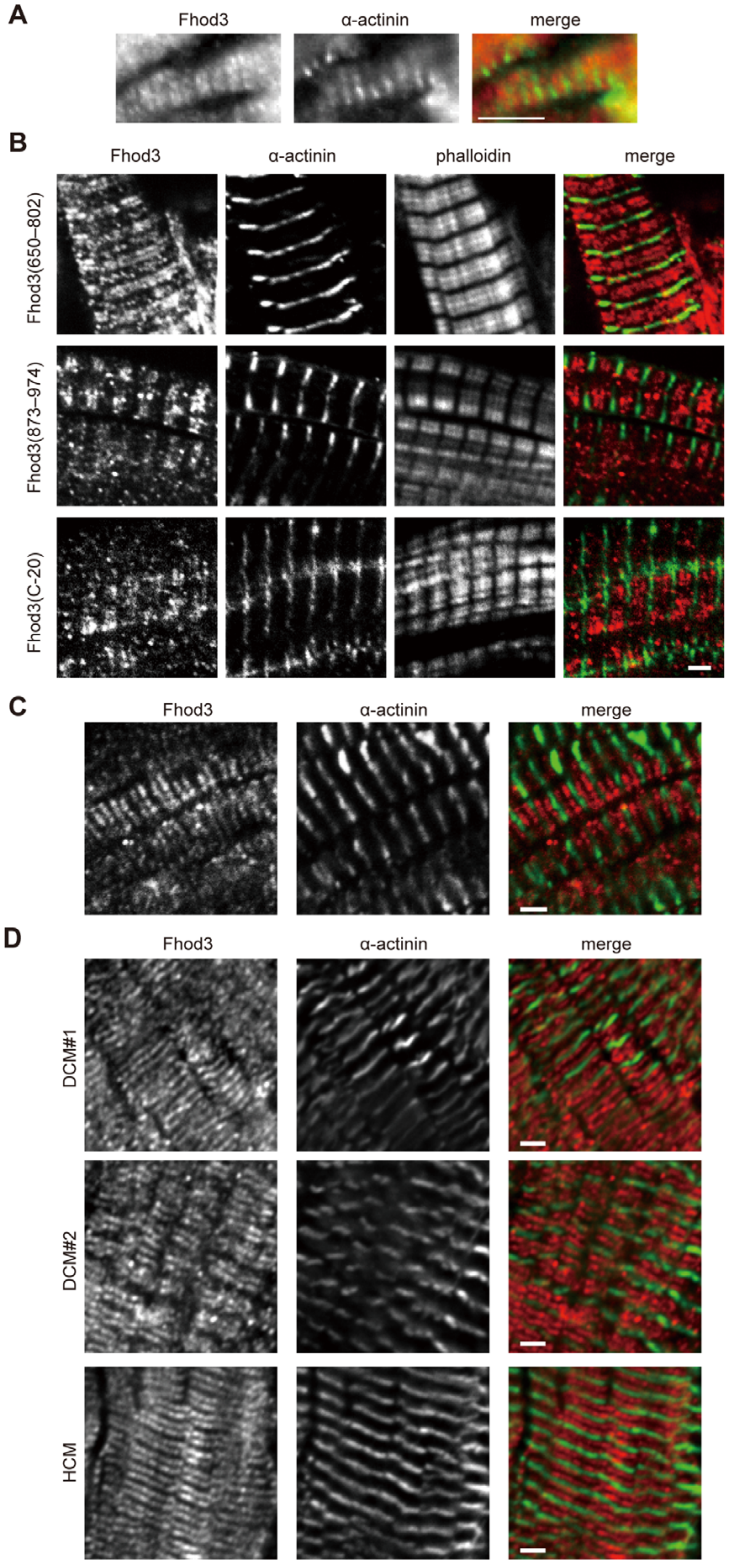
Localization of Fhod3 in the adult heart. (A) Glycerinated fibers from adult mouse hearts were subjected to immunofluorescent double staining for endogenous Fhod3 (red) and α-actinin (green). For Fhod3 staining, the anti-Fhod3-(650–802) polyclonal antibodies were used. Bar, 5 µm. (B) Sections of adult mouse hearts were subjected to immunofluorescent double staining for endogenous Fhod3 (red) and α-actinin (green) followed by phalloidin staining (not shown in merge). For Fhod3 staining, the anti-Fhod3-(650–802), the anti-Fhod3-(873–974), and the anti-Fhod3-(C-20) polyclonal antibodies were used. The anti-Fhod3-(C-20) antibodies used here were pre-adsorbed with an acetone powder of mouse embryonic fibroblast derived from Fhod3 knockout mice. For details, see “Materials and Methods”. Bar, 2 µm. (C) Sections of tissues from a left ventricle of a human heart were subjected to immunofluorescent double staining for endogenous Fhod3 (red) and α-actinin (green). For Fhod3 staining, the anti-Fhod3-(650–802) polyclonal antibodies were used. Bar, 2 µm. (D) Sections of tissues from a left ventricle of two patients (#1 and #2) with idiopathic dilated cardiomyopathy (DCM) and a patients with idiopathic hypertrophic cardiomyopathy (HCM) were subjected to immunofluorescent double staining for endogenous Fhod3 (red) and α-actinin (green). For Fhod3 staining, the anti-Fhod3-(650–802) polyclonal antibodies were used. Bar, 2 µm.

To investigate *in situ* localization of Fhod3 in the sarcomere of the adult heart, we immunostained glycerin-treated fibers from the adult mouse heart with the anti-Fhod3-(650–802) antibodies (Figure 3A). Signals by the antibodies, albeit not very intense, indicated that Fhod3 localizes in the sarcomere in glycerinated muscle fibers. We also examined Fhod3 localization in frozen sections from the adult mouse heart. As shown in Figure 3B, the anti-Fhod3-(650–802) and anti-Fhod3-(873–974) antibodies localized as doublet in the sarcomere in the cryo-sections from adult hearts. The localization of Fhod3 is essentially the same as that in the embryonic heart (Figure 2). Although a substantial proportion of the anti-Fhod3-(C-20) antibodies localizes to the Z-line (Figure S1), signals on the Z-line were selectively attenuated by treatment of these antibodies with an acetone powder of mouse embryonic fibroblasts derived from Fhod3 knockout mice (manuscript in preparation), as shown in Figure 3B (bottom panels). This observation suggests that untreated anti-Fhod3-(C-20) antibodies may contain in part those cross-reacting with a component of the Z-line. Taken together, we conclude that Fhod3 mainly localizes to the middle of the sarcomere not to the Z-line in the adult mouse heart. This appears to be different from a recent observation that a majority of Fhod3 is present at the Z-line in the adult mouse heart [12].

In addition, to examine Fhod3 localization in the human adult heart using the anti-Fhod3-(650–802) antibodies, we prepared frozen sections from a piece of tissue in a noncardiomyopathic left ventricle, which was obtained from a patient with severe aortic stenosis at the time of therapeutic surgery. In the section, Fhod3 localized as doublet in the sarcomere, the boundaries of which were formed by the Z-lines marked with the anti α-actinin antibody (Figure 3C). Thus Fhod3 in the adult human heart likely localizes in the sarcomere in a manner similar to that in the mouse heart. Furthermore, we also tested Fhod3 localization in human hearts with idiopathic dilated cardiomyopathy or idiopathic hypertrophic cardiomyopathy. As shown in Figure 3D, the basic pattern of sarcomere localization of Fhod3 was maintained in these cardiomyopathic hearts.

### Role of the T(D/E)_5_XE region in Fhod3 localization

**Figure 4:**
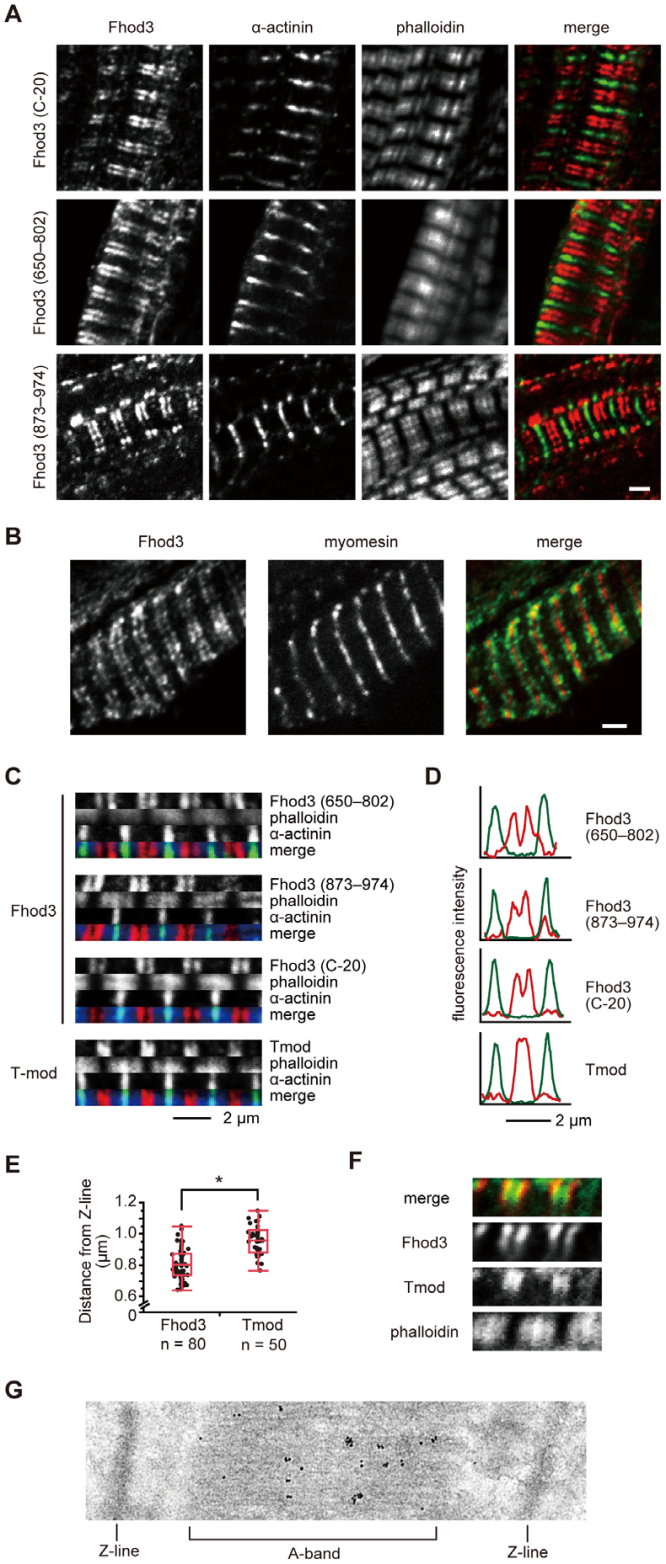
Localization of Fhod3 in the heart of Fhod3 transgenic mice. (A) Sections of adult hearts from transgenic mice that specifically express a high amount of the T(D/E)_5_XE-region-deficient Fhod3 protein in the heart were subjected to immunofluorescent double staining for Fhod3 (red) and α-actinin (green), followed by phalloidin staining. For Fhod3 staining, the anti-Fhod3-(C-20) (top panels), the anti-Fhod3-(650–802) (middle panels), or the anti-Fhod3-(873–974) (bottom panels) polyclonal antibodies were used. Bar, 2 µm. (B) Sections of adult hearts from Fhod3 transgenic mice were subjected to immunofluorescent double staining for Fhod3 (red) and myomesin (green). For Fhod3 staining, the anti-Fhod3-(650–802) polyclonal antibodies were used. Bar, 2 µm. (C) Magnified image of single myofibril from immunostained sections of adult hearts of Fhod3 transgenic mice. Sections were stained for α-actinin (green), phalloidin (blue), and Fhod3 or Tmod1 (red). For Fhod3 staining, the anti-Fhod3-(650–802) (top panels), the anti-Fhod3-(873–974) (2nd panels), and the anti-Fhod3-(C-20) (3rd panels) polyclonal antibodies were used. Bar, 2 µm. (D) Fluorescence intensity profiles in a line scan of sarcomeres. Line scan profiles of fluorescence intensities for the anti-α-actinin antibody (green) and the anti-Fhod3-(C-20) or anti-Tmod antibodies (red) are generated from immunofluorescent images shown in *C*. (E) Distance between the Z-line and Fhod3 and that between the Z-line and Tmod. The distance of the fluorescence peak of Fhod3 or Tmod from that of α-actinin are measured on immunofluorescent images. Box-and-whisker plots indicate 25th percentile (bottom line), median (middle line), 75th percentile (top line), and nearest observations within 1.5 times the interquartile range (whiskers). *, *P*<0.001, Welch's *t* test. (F) Magnified image of single myofibril from immunostained sections of adult hearts of Fhod3 transgenic mice. Sections were stained for Fhod3 (red), Tmod1 (green), and phalloidin (blue). For Fhod3 staining, the anti-Fhod3-(650–802) polyclonal antibodies were used. (G) Ultrastructural localization of Fhod3. Ultrathin cryosections of adult hearts from Fhod3 transgenic mice were immunolabeled using the anti-Fhod3-(650–802) antibodies, and labeling was detected using gold-conjugated secondary antibodies.

When the Fhod3 mRNA containing all the exons except the T(D/E)_5_XE-region-encoding exon 25 is ectopically expressed in cultured cardiomyocytes from neonatal rats, its encoded protein localizes in the sarcomere in the same manner as endogenous cardiac Fhod3, which harbors the acidic region [10], indicating that the T(D/E)_5_XE region is dispensable for Fhod3 localization. On the other hands, it has been reported that, when ectopically expressed in neonatal rat cardiomyocytes, a major population of T(D/E)_5_XE-deficient Fhod3 forms aggregates in the cytoplasm; however, the remainder appears to exist in the middle of the sarcomere, as does the endogenous cardiac isoform [12]. To clarify the role of the T(D/E)_5_XE region in Fhod3 localization *in situ*, we generated transgenic mice that specifically express T(D/E)_5_XE-deficient Fhod3 protein in the heart under the control of the α-myosin heavy chain promoter (Figure S2). Immunohistochemical analysis with the three different anti-Fhod3 antibodies revealed that exogenously expressed T(D/E)_5_XE-deficient Fhod3 localizes as two closely spaced bands in the middle of the sarcomere of the adult heart (Figure 4A). This localization is essentially the same as the *in situ* localization of the T(D/E)_5_XE-containing endogenous cardiac Fhod3 (Fig. 3), indicating that the T(D/E)_5_XE region is dispensable for *in situ* localization of Fhod3 in the sarcomere.

The paired bands stained with the anti-Fhod3 antibodies were well separated from the Z-line, marked with sarcomeric α-actinin (Figure 4A), whereas they were adjacent to the M-line, indicated by the presence of myomesin (Figure 4B). Although Fhod3 localized near the region where the pointed-end-capping protein Tmod existed (Figure 4C), the paired bands of Tmod are closer to each other (*i.e.*, more distant from the Z-line) than those with those of Fhod3, which is indicated by line scan graphs of fluorescence intensity along the long axis of a sarcomere (Figure 4D): the distances from the Z-line marker α-actinin to Fhod3 (0.81±0.10 µm) were significantly shorter than those to Tmod (0.96±0.09 µm) (Figure 4E). Furthermore, co-immunostaining with the anti-Fhod3 and anti-Tmod antibodies confirmed that Fhod3 does not colocalize with the pointed-end capping protein Tmod (Figure 4F). These findings indicate that Fhod3 localizes not to the pointed-ends of thin actin filaments but rather to the zone where actin filaments overlap with myosin filaments.

Furthermore, using sections of the heart prepared from Fhod3 transgenic mice, we examined the ultrastructural localization of Fhod3 in the sarcomere by immuno-electron microscopic analysis. As shown in Figure 4G, gold particles complexed with the anti-Fhod3-(650–802) antibodies were accumulated on the myosin-filament-containing A-band in the middle of the sarcomere. The finding supports the idea that Fhod3 localizes to the zone where actin filaments overlap with myosin filaments.

### Role of the T(D/E)_5_XE region in Fhod3-induced actin organization both *in vivo* and *in vitro*

**Figure 5:**
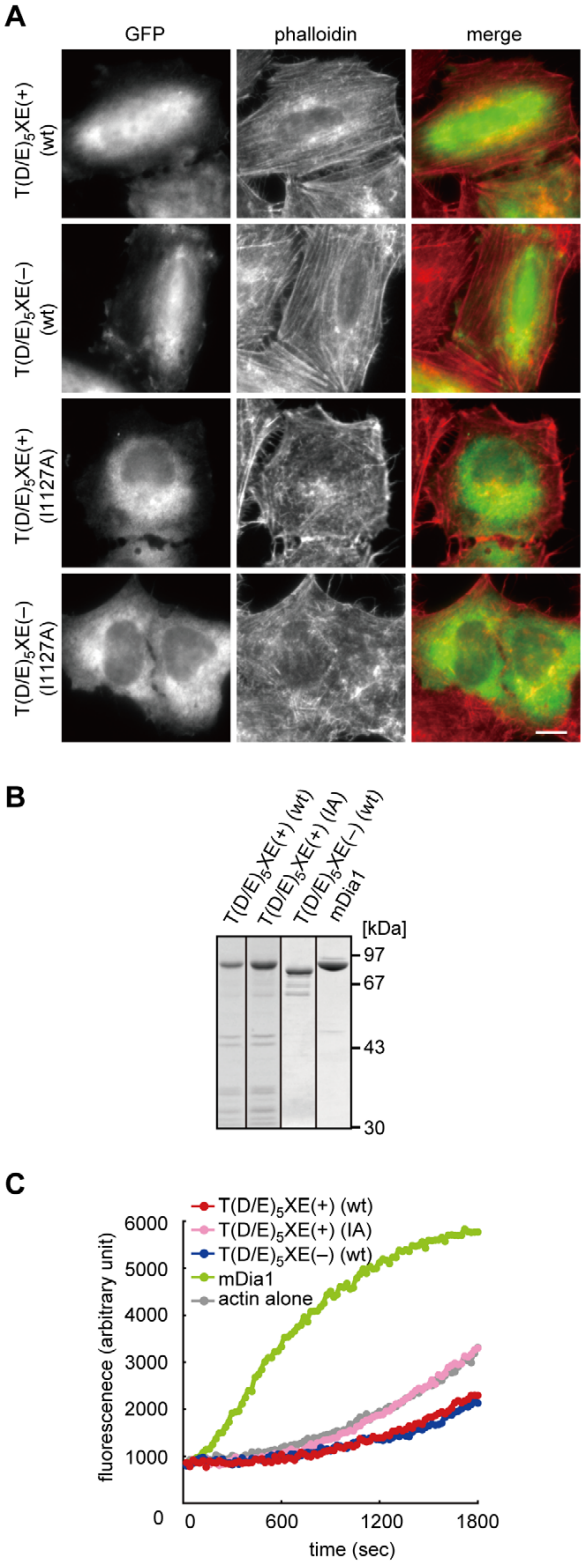
Effect of the T(D/E)_5_XE region of Fhod3 on actin assembly. (A) Effect of the Fhod3 T(D/E)_5_XE region encoded by exon 25 on *in vivo* actin assembly. HeLa cells were transfected with plasmids encoding Fhod3-ΔN (wt) (amino acids 931–1,586) with or without the T(D/E)_5_XE region in the FH2 domain; or plasmids encoding Fhod3-ΔN (I1127A) with or without the T(D/E)_5_XE region. Cells were fixed and visualized by GFP-fluorescence (green) or phalloidin staining (red). Scale bar, 10 µm. (B) SDS-PAGE analysis of purified proteins used in an actin polymerization assay. Purified proteins were subjected to 10% SDS-PAGE and stained with *Coomassie Brilliant Blue*. Positions for marker proteins are indicated in kDa. (C) Effect of the T(D/E)_5_XE region of Fhod3 on *in vitro* actin assembly. G-actin (10% pyrene-labeled) at 2 µM was incubated with 50 nM Fhod3-T(D/E)_5_XE(+)-ΔN (wt), Fhod3-T(D/E)_5_XE(+)-ΔN (I1127A), Fhod3-T(D/E)_5_XE(−)-ΔN (wt), or mDia1-FH1FH2 (amino acids 549–1,175) in the presence of 2 µM profilin I.

To investigate the functional role of the T(D/E)_5_X region in the FH2 domain, we compared the ability of Fhod3 to assemble actin between Fhod3 proteins with and without the T(D/E)_5_XE region. It is known that an N-terminally truncated Fhod3 (Fhod3-ΔN), functioning as a constitutively active form, induces formation of actin stress fibers when expressed in HeLa cells [10]. As shown in Figure 5A, the N-terminally-truncated cardiac Fhod3, which retained the T(D/E)_5_XE region, was as effective as the T(D/E)_5_XE-deleted Fhod3-ΔN in stress fiber formation in HeLa cells, indicating a dispensable role of this region in an *in vivo* actin assembly activity. This is in good agreement with a recent result obtained by quantitative estimation of cellular F-actin content that the T(D/E)_5_XE region has no effect on the general ability of Fhod3 to affect actin polymerization in cells [12]. Furthermore, we tested an *in vitro* ability to assemble actin using purified Fhod3 proteins (Figure 5B). In an *in vitro* actin polymerization assay, Fhod3-ΔN lacking the T(D/E)_5_XE region suppressed spontaneous assembly of actin (Figure 5C), as shown in the previous study [10]. Under the conditions, Fhod3-ΔN including the T(D/E)_5_XE region exerted almost the same effect (Figure 5C). On the other hand, irrespective of the presence or absence of the T(D/E)_5_XE region, Fhod3-ΔN carrying the I1127A substitution, defective in the actin-binding activity [10], did not induce stress fiber formation (Figure 5A). The substitution also resulted in a loss of the *in vitro* actin-organizing activity (Figure 5C). Thus the T(D/E)_5_XE region in the FH2 domain does not appear to be required for actin-organizing activities both *in vivo* and *in vitro*.

## Discussion

In the present study, we show that cardiac Fhod3, containing the regions encoded by the three alternative exons 11, 12, and 25, mainly localizes *in situ* as two closely spaced bands in the middle of the sarcomere in both the developing embryonic heart and the mature adult heart. The bands of Fhod3 exist not on the Z-line but rather adjacent to M-line (Figures 2, 3, 4). Detailed immunohistochemical analysis, in combination with immuno-electron microscopic analysis, demonstrates that, in the sarcomere, Fhod3 localizes not at the pointed ends of thin actin filaments but to a more peripheral zone, where thin filaments overlap with thick myosin filaments (Figure 4). The characteristic localization of Fhod3 in the sarcomere does not appears to require the T(D/E)_5_XE region encoded by exon 25, in contrast to an essential role of exons 11 and 12. The acidic region is also likely dispensable for actin-organizing activities both *in vivo* and *in vitro* albeit it is inserted in the catalytic FH2 domain (Figure 5).

In contrast to the present study, Iskratsch *et al.* have reported that Fhod3 localizes mainly to the Z-line as well as the intercalated-disc in the adult heart [12]. Although the reason for the discrepancy is presently unknown, it might be explicable by the difference in anti-Fhod3 antibodies used. They stained cardiac Fhod3 with affinity-purified polyclonal antibodies raised against the N-terminal region of Fhod3 (amino acid residues 1–339) [12]. On the other hand, we have used three distinct affinity-purified polyclonal antibodies (the anti-Fhod3-(650–802), anti-Fhod3-(873–974), and anti-Fhod3-(C-20) antibodies) raised against their respective regions of amino acid residues 650–802, 873–974, and 1,567–1,586. In some cases, the affinity-purified anti-Fhod3-(C-20) antibodies has been further treated with an acetone powder prepared from Fhod3-deficient cells for complete removal of crossly-reacting antibodies from the monospecific antibody preparation, which treatment appears to emphasize that Fhod3 localizes not to either ends of actin filaments but to the zone where thin actin filaments overlap with myosin filaments in the sarcomere of the adult heart. The discrepancy between the two studies also might be due to the difference in fixation procedures: Iskratsch *et al.* fixed cryosections by immersion with precooled acetone [12], whereas we prepared tissue sections by a perfusion fixation method with 3.7% formaldehyde to preserve the ultrastructure of sarcomeres. In addition, it might be possible that localization of Fhod3 alters depending on the stage of the sarcomere, as does that of the *Drosophila* protein SALS in larval somatic body wall muscles and adult indirect flight muscles: SALS likely localizes at the pointed ends of thin filaments while they are elongating, but it disappears from the pointed ends of full-growth thin filaments and relocates at the region flanking the Z-line [14]. No evidence that Fhod3 localization alters in the heart has been obtained so far, although our present study did not investigate the Fhod3 localization during the elongating stage of the sarcomere.

The exon 25-encoded T(D/E)_5_XE region is not required for the characteristic sarcomere localization of Fhod3 not only in cultured cardiomyocytes but also in the heart of Fhod3 transgenic mice, as shown here. On the other hand, it has been reported that, using neonatal rat cardiomyocytes, a major population of ectopically-expressed T(D/E)_5_XE-deficient Fhod3 forms aggregates in the cytoplasm, although the remainder of this exogenous protein seemingly exists in the middle of the sarcomere as does the endogenous cardiac isoform [12]. It might be possible that, compared with the T(D/E)_5_XE-containing Fhod3, the T(D/E)_5_XE-deficient isoform is unstable but inaccessible to a usual protein degradation pathway, and thus forms cytoplasmic aggregates especially when expressed ectopically at a higher level. The threonine residue of the T(D/E)_5_XE region has been shown to be phosphorylated by casein kinase 2; the phosphorylation likely prevents formation of aggregates, which are recruited to autophagosomes [12].

In the sarcomere of the heart, Fhod3 localizes to neither end of thin actin filaments but to the region where actin filaments overlap with thick myosin filaments. On the other hand, Fhod3 likely organizes F-actin assembly via the FH2 domain by associating with the barbed end, in a manner similar to that by other formin family proteins [10]. The functional link with the characteristic localization of Fhod3 in the sarcomere is presently unknown, which should be clarified in future studies.

## Materials and Methods

### Ethics Statement

Procedures using human samples were conducted in accordance with the Declaration of Helsinki and approved by the Kyushu University Institutional Review Board for Clinical Research (Permit Number: 22-170). We obtained written informed consent from all the participants. All procedures using mice were performed in strict accordance with the guidelines for Proper Conduct of Animal Experiments (Science Council of Japan). The experimental protocol was approved by the Animal Care and Use Committee of Kyushu University (Permit Number: A22-005-1). All efforts were made to minimize the number of animals used and their suffering.

### Plasmids

The cDNA fragments encoding mouse Fhod3 of 1,578 amino acids (designated as Fhos2L in [11]), which contains all the 28 exons except exon 25, was prepared as previously described [11]. The cDNA fragments encoding mouse Fhod3 of 1,586 amino acids, which contains all the 28 exons, was cloned by RT (reverse transcriptase)-polymerase chain reaction (PCR). The cDNAs for Fhod3-ΔN, lacking the N-terminal 930 amino acids, were constructed by PCR using the cDNAs encoding mouse Fhod3 with or without exon 25. The cDNA for mDia1-FH1FH2 (549–1,175) was prepared as described previously [10]. Mutations leading to the indicated amino acid substitutions were introduced by PCR-mediated sited-directed mutagenesis. The DNA fragments were ligated to pEGFP-C1 (Clontech) or pEF-BOS for expression in HeLa cells as an N-terminally green fluorescent protein (GFP)–tagged protein or for expression in HEK-293F cells as an N-terminally FLAG–tagged protein, respectively. All the constructs were sequenced for confirmation of their identities.

### Antibodies

Rabbit polyclonal antibodies specific for Fhod3 (anti-Fhod3-(650–802), anti-Fhod3-(873–974), and anti-Fhod3-(C-20) antibodies) were prepared as previously described [11]. Briefly, the anti-Fhod3-(650–802) and anti-Fhod3-(873–974) antibodies were raised against the respective GST–fusion proteins comprising amino acid residues 650–802 and 873–974 of Fhod3; the anti-Fhod3-(C-20), was raised against a synthetic peptide corresponding to the C-terminal 20 amino acids (1,567–1,586) of Fhod3. These anti-Fhod3 antibodies were affinity purified by a HiTrap-NHS column (Amersham Bioscience) conjugated with MBP–fusion proteins corresponding to the immunizing regions or with the synthetic peptide used for immunization, respectively. In the case of staining of sections from hearts of adult mice, anti-Fhod3-(C-20) antibodies were pre-adsorbed with an acetone powder of mouse embryonic fibroblasts derived from Fhod3 knockout mice (Acc. No. CDB0598K: http://www.cdb.riken.jp/arg/mutant%20mice%20list.html). The details of the Fhod3 knockout mice will be reported elsewhere. Immunofluorescent images using unadsorbed anti-Fhod3-(C-20) antibodies without pre-adsorption are shown in Figure S1. The specificity of the anti-Fhod3-(650–802) antibodies was confirmed by immunoblot analysis using cardiac tissue lysates (5 µg of protein) from *Fhod3* knockout mouse embryos at E10.5 or from heterozygous *Fhod3^+/−^* mouse embryos at E10.5 (Figure S3). The mouse monoclonal antibody against α-actinin (clone EA-53) was purchased from Sigma-Aldrich; the goat polyclonal antibodies against myomesin-1 (C-16) from Santa Cruz; the rabbit polyclonal antibodies against Tmod1 from ProteinTech Group; the mouse polyclonal antibodies against Tmod1 from Abnova; the fluorescent secondary antibody conjugated with Alexa Fluor 488 or 594 against mouse IgG, rabbit IgG, or goat IgG from Invitrogen; and Alexa Fluor 555-conjugated F(ab')_2_ fragment of anti-rabbit IgG from Cell Signaling Technology.

### RT-PCR analysis

The expression of the Fhod3 mRNAs was determined by RT-PCR using total RNA as a template, which was extracted from the adult (3 weeks old) or embryonic (E17.5) C57BL/6 mice tissues using TRIzol reagent (Invitrogen). Primers were designed on the basis of the mouse sequence reported [11], [12]. For detection of exon 25, the following primers were used: ‘S6’, 5′- GGGAAGATGATCACTGACTCT-3′ (a forward primer specific for Fhod3 mRNAs lacking exon 25); ‘S7’, 5′- GACGAGGATGAAGCTGAGTCT-3′ (a forward primer specific for Fhod3 mRNAs containing exon 25); ‘S8’, 5′- AGAGGGAAGATGATCACTGAC-3′ (a common forward primer for both isoforms); and ‘RV3’, 5′-ATCAGTGACACTAGGTGACTC-3′ (a common reverse primer for both isoforms). The PCR fragments were subjected to 1% agarose gel electrophoresis. For sequencing analysis of exon 25, PCR fragments amplified using the two unique oligonucleotide primers 5′-cagggatccGTGGAAAACTTTCCGGACAGC-3′ and 5′-cgtgaattcACATGTTCTCATGTTCGGCTGC-3′ (sequences from the cDNA are capitalized) were subcloned into pEF-BOS [15]. More than ten different inserts were sequenced for the indicated tissue. For detection of exons 11 and 12, PCR analysis was performed using a forward primer 5′-cagggatccGTCAAACCCTGGTCCAACATC-3′ and a reverse primer 5′-gaactcgagtcaCCTGAACGAGGATGTGAGAAG-3′ (sequences from the cDNA are capitalized) [11].

### Mice

Transgenic mice expressing Fhod3 that contained all the exons except exon 25 under the control of the α-myosin heavy chain promoter [16] were generated on a C57BL/6 background. Six different founder lines showing different level of Fhod3 expression in the heart were obtained without juvenile morbidity or mortality. Among these lines, hemizygotes of line 4 showing minimal expression of Fhod3 protein (Figure S2) were used for immunofluorescent microscopic analysis, and hemizygotes of line 6 showing maximal expression of Fhod3 protein were used for immuno-electron microscopic analysis. All mice were kept in a specific pathogen-free animal facility at Kyushu University. All procedures using mice were performed in strict accordance with the guidelines for Proper Conduct of Animal Experiments (Science Council of Japan). The experimental protocol was approved by the Animal Care and Use Committee of Kyushu University (Permit Number: A22-005-1). All efforts were made to minimize the number of animals used and their suffering.

### Human tissue preparation

Human heart tissue of a hypertrophic left ventricle was obtained at the time of therapeutic surgery from a patient that suffered from cardiac failure due to severe aortic stenosis. Cardiomyopathic tissues of left ventricles were obtained at the time of therapeutic surgery from two patients with idiopathic dilated cardiomyopathy (DCM) and a patient with idiopathic hypertrophic cardiomyopathy (HCM). Myocardial tissues without apparent fibrosis were subjected to immunofluorescence microscopy. Procedures using human samples were conducted in accordance with the Declaration of Helsinki and approved by the Kyushu University Institutional Review Board for Clinical Research (Permit Number: 22-170). We obtained written informed consent from all the participants. The dissected pieces of human heart tissue were frozen in isopentane precooled with liquid nitrogen. Cryosections (5 µm thick) were fixed in 3.7% formaldehyde and subjected to immunofluorescence staining.

### Mouse tissue preparation

C57BL/6 mice or transgenic mice, aged 3–4 weeks, were deeply anesthetized with an intraperitoneal injection of pentobarbital (50 mg/kg body weight). After exposure of the heart, 5 ml of PEM buffer (1 mM EGTA, 1 mM MgCl_2_, and 100 mM PIPES, pH 6.9) containing 20 mM KCl was administered from the left ventricle, followed by the administration of 10 ml of 3.7% formaldehyde in phosphate-buffered saline (PBS; 137 mM NaCl, 2.68 mM KCl, 8.1 mM Na_2_HPO_4_, and 1.47 mM KH_2_PO_4_, pH 7.4). For facilitation of selective coronary perfusion, the ascending aorta was clamped with a hemostat and the right atrium was clipped with surgical scissors before the perfusion. For preparation of the embryonic samples, timed pregnant mice were sacrificed via cervical dislocation, and E15.5–16.5 embryos were dissected from the uterus and anesthetized by hypothermia [17]. The hearts of embryos were exposed under a dissecting microscope. After clamping of the ascending aorta and clipping of the right atrium, 1 ml of PEM buffer containing 20 mM KCl was administered from the left ventricle with a pulled capillary tube, followed by perfusion of 3 ml of 3.7% formaldehyde. The fixed hearts were removed from the deceased mice, cut into small pieces, and immersed for 90 min at 4°C in the same fixative. The tissues obtained were washed in PBS, subjected to osmotic dehydration in 30% sucrose overnight at 4°C, and embedded in OCT compound (Sakura). The blocks were frozen at −80°C and cut into 5 µm sections using a cryostat (Thermo Scientific HM550). The sections were air dried and stored at −80°C before staining.

### Primary culture of mouse embryonic cardiomyocytes

Primary cardiomyocytes were prepared from embryonic hearts of C57BL/6 mice according to the method of Goshima [18] with minor modifications. Briefly, hearts were isolated from E14.5–16.5 embryos, dissected and then digested with collagenase type II (300 units/ml, Worthington CLS2) for three successive periods of 10 min each at 37°C. Cells were preplated for 70 min into 90-mm culture dishes in DMEM supplemented with 10% FCS to reduce the number of non-myocytes. Unattached cells were plated and cultured in DMEM with 10% FCS.

### Immunofluorescence staining

Sections were washed with PBS containing 0.1% Triton X-100, and blocked with a blocking buffer (PBS containing 3% bovine serum albumin and 2% goat serum) for 2 h at room temperature. Sections were labeled with primary antibodies diluted overnight at 4°C in the buffer containing 0.1% Triton X-100, washed in PBS containing 0.1% Triton X-100, and then labeled for 1 h at 37°C with a fluorescein-conjugated secondary antibody mixture in the same buffer containing Alexa-647-phalloidine (Invitrogen) for F-actin staining. In the case of Fhod3 staining in tissue sections, F(ab')_2_ fragments of anti-rabbit IgG was used. Cultured cardiomyocytes were fixed in 3.7% formaldehyde and immunostained as previously described [10]. Images shown in Figure 2C, Figure 3B, Figure 3C, and Figure 4 (A–D) were taken with an LSM510 confocal scanning laser microscope (Carl Zeiss MicroImaging), and images shown in Figure 2A, Figure 2B, Figure 3A, and Figure 5A were with an Axiovert 200 microscope (Carl Zeiss MicroImaging) coupled to an Axiocam HRm camera (Carl Zeiss MicroImaging).

### Immuno-electron microscopic analysis

Immuno-electron microscopy of ultrathin cryosections was performed by the method of Tokuyasu [19] with minor modifications. Briefly, the perfusion-fixed hearts of transgenic mice expressing Fhod3 were cut into small pieces and immersed in 0.1 M sodium cacodylate containing 4% paraformaldehyde and 0.4% glutaraldehyde for 60 min at 4°C. After washing with PBS, the tissues were cryoprotected in 2.3 M sucrose overnight at 4°C, and frozen in liquid nitrogen. Ultrathin cryosections (100 nm thick) were cut using a Leica EM UC7/FC7 cryo-ultarmicrotome and sections were collected onto 100 mesh formvar/carbon-coated nickel grids. After washing, grids were blocked in 3% BSA and 2% goat serum, and incubated overnight with the anti-Fhod3-(650–802) antibodies and subsequently incubated for 2 h with anti-rabbit IgG antibody conjugated with 10-nm gold particle (EY lab). After washing, grids were post-fixed with 2.5% glutaraldehyde and embedded in a mixture containing 2% polyvinyl alcohol and 0.2% uranyl acetate. Sections were analyzed on a Tecnai-20 transmission electron microscope (FEI Corp.), and images were collected with an Eagle 2k HR digital camera (FEI Corp.). The primary antibodies-specific labeling was confirmed by control experiments in which primary antibodies are omitted.

### *In vivo* actin assembly assay

HeLa cells were transfected using Lipofectamine (Invitrogen) with a plasmid for Fhod3-ΔN (wt) (amino acids 931–1,586) with or without the T(D/E)_5_XE region or one for Fhod3-ΔN (I1127A) with or without the T(D/E)_5_XE region, and cultured for 3 h. After the addition of DMEM containing 10% fetal calf serum (FCS), cells were cultured for another 13 h. After washing with PBS, cells were fixed for 15 min in 3.7% formaldehyde, permeabilized for 4 min with 0.1% Triton X-100 in PBS, and blocked for 60 min with PBS containing 3% BSA. For F-actin staining, Texas Red-X phalloidin (Invitrogen) was used. Images were taken with an Axiovert 200 microscope (Carl Zeiss MicroImaging) coupled to an Axiocam HRm camera (Carl Zeiss MicroImaging).

### *In vitro* actin polymerization assay

Fhod3 and mDia1 proteins for an actin polymerization assay were prepared as described previously [10]. Briefly, FreeStyle HEK-293F cells (Invitrogen) were transfected with an expression vector encoding the cDNA for Fhod3 or mDia1. The transfected cells were broken with the lysis buffer, and the lysate was precipitated with an anti-FLAG antibody (M2)-conjugated agarose (Sigma-Aldrich). Proteins eluted with FLAG-peptide (200 µg/ml) in X buffer (2 mM MgCl_2_, 100 mM KCl, 0.1 mM CaCl_2_, 5 mM EGTA, 1 mM DTT, and 10 mM Hepes, pH 7.9) were used for an actin polymerization assay as described below. Human profilin I were expressed in *E.coli* as a glutathione *S*-transferase (GST) fusion protein, purified by glutathione-Sepharose 4B (GE Healthcare), and cleaved with PreScission protease (GE Healthcare). Pyrene-actin polymerization assays were performed in X buffer as described previously [10]. Briefly, G-actin (10% pyrene-labeled) was prepared in G buffer (0.2 mM CaCl_2_, 0.2 mM ATP, 0.2 mM DTT, and 5 mM Tris-HCl, pH 8.0). Polymerization reactions were performed in 100 µl of X buffer containing 2 µM actin (10% pyrene-labeled), 2 µM profilin I, and 50 nM Fhod3-ΔN or mDia1-FH1FH2 (amino acids 549–1,175). All reaction components except the actin were mixed in X buffer, and the reaction was started by the addition of actin. Fluorescence changes (excitation wavelength of 365 nm; emission wavelength of 407 nm) were measured using the multilabel plate reader EnSpire (Perkin Elmer).

### Immunoblot analysis

Immunoblot analysis was performed as previously described [10], [11]. Briefly, hearts of mice were homogenized and sonicated at 4°C in a lysis buffer (10% glycerol, 135 mM NaCl, 5 mM EDTA, and 20 mM Hepes, pH 7.4) containing Protease inhibitor cocktail (Sigma-Aldrich). The lysates were applied to SDS-PAGE and transferred to a polyvinylidene difluoride membrane (Millipore). The membrane was probed with the anti-Fhod3-C20 antibodies, followed by development using ECL-plus (GE Healthcare) for visualization of the antibodies.

## Supporting Information

Figure S1 **Localization of Fhod3 in the adult heart.** Sections of adult mouse hearts were subjected to immunofluorescent double staining with the untreated anti-Fhod3-(C-20) antibodies (red) and the anti-α-actinin monoclonal antibody (green) followed by phalloidin staining. Bar, 2 µm.
(PDF)

Figure S2 **Cardiac expression of Fhod3 in the transgenic mice expressing Fhod3.** Cardiac tissue lysates (5 µg of protein) from transgenic mice (Tg) aged 3 weeks or non-transgenic mice (Non-Tg) aged 3 weeks were analyzed by immunoblot with the anti-Fhod3-(C-20) antibodies.
(TIF)

Figure S3 **Characterization of the affinity-purified polyclonal antibodies against Fhod3.** Lysates of cardiac tissues (5 µg of protein) from *Fhod3* knockout mouse embryos at E10.5 or heterozygous *Fhod3^+/−^* mouse embryos at E10.5 were analyzed by immunoblot with the anti-Fhod3-(650–802) and anti-GAPDH antibodies.
(PDF)
