# Supplementary figures and images for: Expression and Subcellular Localization of Mammalian Formin Fhod3 in the Embryonic and Adult Heart
Source: PLoS One. 2012 Apr 11;7(4):e34765. doi: 10.1371/journal.pone.0034765 (PMC3324543; doi:10.1371/journal.pone.0034765)

Figure S1 (Kan-o et al.)

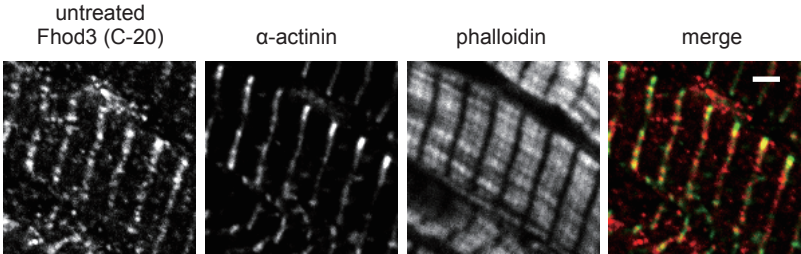

Supplement: Figure S1 — Localization of Fhod3 in the adult heart. Sections of adult mouse hearts were subjected to immunofluorescent double staining with the untreated anti-Fhod3-(C-20) antibodies (red) and the anti-α-actinin monoclonal antibody (green) followed by phalloidin staining. Bar, 2 µm. (PDF) [file pone.0034765.s001.pdf]

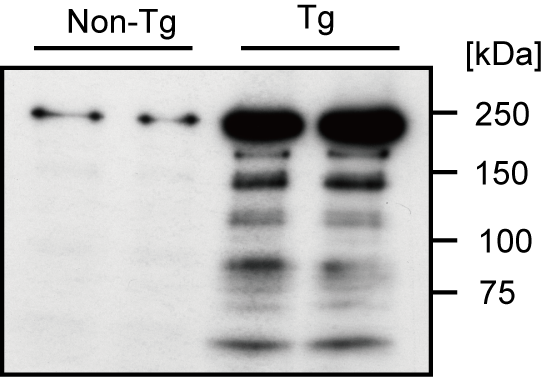

Supplement: Figure S2 — Cardiac expression of Fhod3 in the transgenic mice expressing Fhod3. Cardiac tissue lysates (5 µg of protein) from transgenic mice (Tg) aged 3 weeks or non-transgenic mice (Non-Tg) aged 3 weeks were analyzed by immunoblot with the anti-Fhod3-(C-20) antibodies. (TIF) [file pone.0034765.s002.tif]

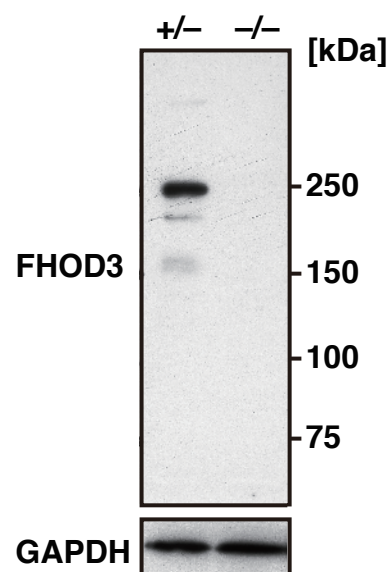

Supplement: Figure S3 — Characterization of the affinity-purified polyclonal antibodies against Fhod3. Lysates of cardiac tissues (5 µg of protein) from Fhod3 knockout mouse embryos at E10.5 or heterozygous Fhod3+/− mouse embryos at E10.5 were analyzed by immunoblot with the anti-Fhod3-(650–802) and anti-GAPDH antibodies. (PDF) [file pone.0034765.s003.pdf]
